# PReS-FINAL-2293: An attempt to analyze variants of systemic lupus erythematosus onsets in childhood

**DOI:** 10.1186/1546-0096-11-S2-P283

**Published:** 2013-12-05

**Authors:** E Kuchinskaya, M Dubko

**Affiliations:** 1 Saint-Petersburg State Pediatric Medical University, Saint-Petersburg, Russian Federation

## Introduction

Childhood-onset systemic lupus erythematosus (SLE), compared with adult-onset variants, is characterized by severity, variety of organ involvement, unpredictable history and diversity of onset variants.

## Objectives

To analyze and try to systemize variants of SLE onsets among patients of rheumatology department.

## Methods

We collected and analyzed 27 cases of systemic lupus erythematosus in children (age up to 18 years) admitted to our department from 2006 to 2013.

## Results

Among 27 patients there were 21 (78%) females and 6 (22%) males; 11 years was an average age of onset. Variants of SLE onset can be systemized by different ways:

## According to time from first manifestation to complete clinical presentation

«Acute» onset: time from first symptom to complete clinical presentation is less than 1 month - 9% (14 of 27) Among such cases were 4 (14,8%) with delayed (5-14 months) new organ involvement. 3 patients presented with renal involvement: 2 cases of microhematuria, proteinuria in patients without cytostatic therapy; 1 case of nephrotic syndrome in patient treated by chloroquine. 2 patients (treated by cytostatic drugs) presented with neuropsychiatric manifestations: seizures, focal cortical neurologic signs.

«Subacute» onset: time from first symptom to complete clinical presentation - from 1 to 3 months - 14,8% (4 of 27). This variant is characterized by progressive, step-by-step involvement of new organ systems without any regularity in priority.

«Prolonged» onset: slow evolution of clinical manifestations (from 8 to 24 months in this research) - 33,3% (9 of 27)

## According to inflammatory activity of disease onset

55% of 27 patients presented fever (17 of 27). In 66% cases increased ESR/CRP rate was detected. Fever is more common for patients with «acute» or «subacute» onset variant (61% - 11 of 18), all of them also had increased ESR/CRP rate. Fever also presented in 4 patients of 9 «prolonged» onsets (44%), increased ESR/CRP rate in 7 of 9 (77%). Both fever and increased ESR/CRP rate were detected in different time from onset.

## According to primary affected organ system

Dermatologic manifestation (malar rash, photosensitivity, non-specific rash) was the first symptom for majority of patients (44% -12 of 27), 3 of them had both dermatologic manifestation and oral ulcers. 7 patients (25,9%) started their history with different autoimmune cytopenias, including acute haemolytic anemia as the first symptom (2 cases), and acute thrombocytopenic purpura (3 cases). Frank arthritis or arthralgia presented in 7 cases (25,9%) as single symptom or in combination with another manifestations. 3 cases started with thrombosis as manifestation of antiphospholipid syndrome (later confirmed by laboratory tests). There was 1 case with episodes of severe headaches as single complaint for a long time, 1 case with vein thrombosis of leg; one patient with appendicitis and intestinal ulcers (as manifestation of mesenterial vein trombosis). 2 patients had prolonged history (up to 2 years) of non-specific manifestations (fatigue, weakness, weight loss, myalgia) before first clinical symptoms.

## Conclusion

It's hard to systemize onsets of systemic lupus erythematosus because of their variability; and this variability causes variations of disease management and treatment tactics.

## Disclosure of interest

None declared

